# Use of EQA for assessing variation in post-analytical interpretation of results using a clinical case of ?adrenal insufficiency taking into consideration variation in analytical performance of cortisol

**DOI:** 10.3389/bjbs.2026.16559

**Published:** 2026-07-29

**Authors:** Rachel Marrington, Michelle Anderson, Martin Roch, Sarah Davies, Rosie Forster, George Williams, Johnathan d’Oyen-Fitchett, Finlay MacKenzie

**Affiliations:** Birmingham Quality (Member of the UK NEQAS Consortium), University Hospitals Birmingham NHS Foundation Trust, Birmingham, United Kingdom

**Keywords:** adrenal insufficiency, cortisol, EQA, post-analytical, short synacthen test

## Abstract

**Introduction:**

Laboratory medicine is evolving at a rapid rate, not only in terms of the repertoire of tests available and automation of the laboratory and procedures, but also in how laboratory data is used within “calculated tests,” algorithms and guidelines. The goal is diagnostic and treatment improvements for the patient; however, there are real patient safety concerns if the limitations of what is often described as the “simple blood test” are not sufficiently understood and appreciated. The aim of this study was to investigate how laboratories take into consideration variation in cortisol assays when managing potential cases of adrenal insufficiency when they had a single cortisol measurement. For English laboratories also assessed compliance to NICE Guidance NG243 — “Adrenal insufficiency: identification and management,” published in 2024.

**Materials and methods:**

The UK NEQAS for Steroid Hormones Scheme provides an External Quality Assessment (EQA) service for Cortisol. In December 2025, three serum specimens were distributed for cortisol analysis and interpretation based on a provided scenario — each specimen taken from a 25-year-old female at 08:30, clinical details — ?Adrenal insufficiency. Participants were required to interpret results into three categories — (1) Person may have adrenal insufficiency, (2) Probability of adrenal insufficiency uncertain or (3) Adrenal insufficiency very unlikely. Data has been evaluated based on the reported cortisol result and corresponding interpretation, broken down by manufacturer and location of laboratory.

**Results:**

Cortisol results were returned by ∼370 Participants with ∼300 interpretating their result. Specimen 536A had a cortisol of 122 nmol/L (SD 9 nmol/L, %CV 7.5%), 536B — 307 nmol/L (SD 24 nmol/L, %CV 7.8%) and 536C — 260 nmol/L (SD 19 nmol/L, %CV 7.2%) based on a mass spectrometry method mean (validated by a candidate reference method). According to NG243, these cortisol results should correspond to Codes (1), (3) and (2) respectively. As expected, there was variation in the cortisol result reported and the subsequent interpretation.

**Discussion:**

This study utilises EQA to look at the variation in cortisol measurement specifically in the context of identifying adrenal insufficiency. By linking post-analytical interpretation to the analytical measurement we have identified variation in both cortisol assays and interpretation of results.

## Introduction

### How laboratory data is used

According to the Royal College of Pathologists, 300,000 tests are performed every working day in England, with 95% of clinical pathways relying on patients having access to pathology services [1]. Laboratory data is used to support decision-making and ensure patient safety across many fields.

Laboratory tests are *In Vitro* Diagnostics (IVD) which are tests that analyse samples outside of the body, such as the general public might call “blood tests.” Test results are widely used for the diagnosis and monitoring of disease. Automation has significantly transformed laboratory testing over the last decade by streamlining processes at a time when workload has been rapidly increasing. The role of the diagnostic laboratory has always been more than merely the provision of an analytical result for a test that a clinician has requested. As well as ensuring the accuracy of the result, the laboratory provides service users information to help guide requesting (pre-analytical) and interpretation of the result (post-analytical).

Though a test result is mainly thought to impact patient management at that current point in time, results may be used as part of algorithmic tests in the future, for example the Acute Kidney Injury (AKI) algorithm which uses creatinine results over the last year to aid determination of the correct AKI stage [2]. Monitoring of disease requires the review of serial laboratory test measurements to look for clinically significant changes which can then be used to adjust treatment and provide individualised care. For this to be effective clinicians need to have confidence in the reliability of results. Clause 7.4.1.6 (f) of ISO 15189:2022 states that reports should *identify the examination method used, where relevant, including, where possible and necessary, harmonised (electronic) identification of the measurand and measurement principle* [3]. This is particularly important for the monitoring of tumour marker results especially when patients may have blood sent to different laboratories as part of their routine care.

Test results are not always about the person who is being tested, many patients are also part of clinical trials with the results impacting many more patients into the future. Clinical trials may be multicentre clinical trials with sample analysis undertaken at different laboratories which may use different analysers/methodologies. For meaningful results and conclusions there needs to be harmonisation of results between all sites. This has been recognised in a comparative study which recommended harmonisation efforts should be undertaken in multicentre trials to ensure accurate data analysis [4].

Storage of laboratory data in large datasets, on the “Cloud,” is allowing biomedical research to be upscaled and allows international collaboration. “Big data” allows integration of clinical and biomedical data from many sources. Harmonisation of results and stability of methods is crucial to allow this data to be used effectively. Without this, incorrect conclusions on data may be formed, or crucial discoveries may be missed.

### Guidelines and “magic numbers”

The management and treatment of patients is routinely guided by local and national guidelines. The most commonly used in the UK include NICE guidelines which are evidence-based recommendations designed to improve health and social care in England and Wales with an aim to ensure a consistent approach to treatment and care, helping to reduce variation in healthcare quality [5]. The Scottish Intercollegiate Guidelines Network (SIGN) is used in Scotland [6]. NICE and SIGN work with healthcare professionals to develop guidance; however, this doesn’t always include the laboratory and doesn’t always take into consideration any manufacturer/assay related bias. Other society specific guidelines and protocols are also used which may not include manufacturer/assay related bias (likely to be due to lack of awareness) especially if looking at evidence from a small subset of laboratories that are using a single provider of analytical equipment.

It is common that guidelines involving pathology tests include “harsh” cut-offs either for classification of a patient, or as part of a diagnostic pathway. Examples of these so called “magic-numbers” that are commonly used include having a HbA1c of 48 mmol/mol for diagnosis of Diabetes as defined by the WHO in their report for the use of glycated haemoglobin (HbA1c) in diagnosis of diabetes mellitus [7]. Another example is the Fibrosis (FIB)-4 score, where although NICE recommends a single score of greater than 2.67 suggests advanced liver fibrosis; however, there is no consideration whether the ALT and AST methods used in the calculation should be those that the IFCC recommend and which do contain pyridoxal-5-phosphate or whether non-IFCC methods can be used [8]. There are significant differences between the IFCC recommended and non IFCC recommended assays for ALT and AST results in patients with liver disease which consequently does impact the FIB-4 result [9].

Most if not all renal pathways incorporate serum creatinine into either (i) an equation — estimated glomerular filtration rate (eGFR) and Kidney Failure Risk Equation (KFRE) for Chronic Kidney Disease (CKD) [10] — or (ii) or part of an algorithm — AKI [11]. It is well known that there are differences between the performance of the Jaffe creatinine and Enzymatic creatinine assay, and even differences exist between manufacturers within each method principle; however, as of 2026 ∼15% of the UK are still using the Jaffe creatinine assay *(data from the UK NEQAS for Acute and Chronic Disease Scheme January 2026)* [12].

### NICE guideline NG243

“Adrenal insufficiency: identification and management” is the example that is used in this paper. NG243 covers identifying and managing adrenal insufficiency (hypoadrenalism) in babies, children, young people and adults [13]. Table 1 in NG243 provides cortisol cut-offs for the interpretation of serum cortisol levels from an 8 am–9 am test. There is a rider that the *“cut-offs are only for use with modern immunoassays. Local guidelines may need to be followed if alternative assays are used.”* There is no definition of what a modern immunoassay is. The majority of laboratories are likely to be correct in thinking that using an automated analyser is using a modern immunoassay rather than a manual solvent extraction method or even the historical fluorometric Mattingly assay [14]. NICE Guidelines do apply to the UK, but specifically England. Decisions on how NICE Guidelines are implemented in the devolved nations are up to relevant government ministers. It is not mandatory to apply the recommendations, and the guideline does not override the responsibility to make decisions appropriate to the circumstances of the individual.

**TABLE 1:** Example comments provided by participants at Distribution 536 specifically related to cortisol and the interpretative exercise.

Difficult to interpret without further clinical information — e.g. symptoms, stress?, exogenous glucocorticoids? Or elevated oestrogens? (pregnancy, OCP)
Suggest short synacthen test for all these samples
We interpret based on request location, clinical details, and other results. For example, for a GP patient with a morning cortisol of 266 nmol/L who is generally well and has normal electrolytes, we’d say the result makes adrenal insufficiency fairly unlikely. For a patient acutely admitted with hyponatraemia or relevant signs/symptoms, we’d say that cortisol of 310 nmol/L requires further investigation for possible adrenal insufficiency. We’ve seen cases of adrenal crisis with cortisol >400 nmol/L
Need to consider context to ensure that adrenal insufficiency is not missed in acutely unwell patient
Please note oral oestrogens increase total cortisol therefore normal cut offs do not apply For patients not taking oral oestrogens for an early morning cortisol level (between 8 and 9 am) <150 nmol/L is suspicious of adrenal insufficiency. Suggest referral to endocrinology 150–300 nmol/L the probability of adrenal insufficiency is uncertain. Consider repeating 8–9 am serum cortisol. If it remains at this level, seek endocrinology advice or referral >300 nmol/L makes adrenal insufficiency very unlikely
Results interpreted according to all wales guidance. NICE guidance not used for interpretation of cortisol results

### Guidelines in use in other nations

There are no equivalent Scottish Intercollegiate Guidelines Network (SIGN) for adrenal insufficiency; however, the Scottish Paediatric Endocrine Group (SPEG) does provide guidance for assessment of adrenocortical function [15]. Interpretation is based on the cortisol concentration at 60 min (>430 nmol/L Abbott assay). We are not aware of specific adrenal insufficiency guidance for Wales and Northern Ireland; however, local protocols may exist.

Result interpretation for adrenal insufficiency has been covered by one similar exercise (Distribution 527 (March 2025) — which focused on short synacthen test interpretation in adult males. Target audits for short synacthen tests have also looked at variation in practice across the UK [16].

### Accreditation to ISO 15189:2022

Section 7.3 of ISO 15189:2022 focuses on Examination processes and there is one key area which is important for how laboratory data is used in the context discussed thus far [3]. Section 7.3.7.4 looks at the comparability of examination results and ensuring that a process is in place within a given laboratory. The aim is to assess the comparability of results for examinations (analysis) that occurs on different methods or equipment, at different sites. If differences are observed the impact of these differences on biological reference intervals and clinical decision limits should be evaluated and acted upon with users informed of any clinically significant differences in the comparability of results. In reality this is probably only limited to different methods and/or different equipment and/or on multiple sites. However, service users including clinicians need to be aware of all limitations for all methods that could impact comparability and interpretation of results for any protocols and guidelines that they are working to, which is especially important if clinicians are using data as part of research to develop new protocols.

This is where EQA is of significant value. EQA is the retrospective assessment of the performance of an assay system usually undertaken by the analysis of physical clinical specimens. By careful scheme design a good EQA scheme can probe many areas of an assay system, not only the analytical performance of an assay. EQA specimens should be as close in matrix as possible to a patient sample which ensures that it mimics the characteristics of patient material when assayed. Laboratories like to compare themselves with other users of the same method; however, this is not good practice and is not always appropriate. The Target or Assigned Value should always be the “best estimate of the truth.” This may be from a Reference Method, or from a validated target value within the data in the Scheme. Though it is likely that an assay in an individual laboratory can only perform as well as other users of the method, this may not be good enough for the patient [17–23].

### How EQA can be used

Clause 7.3.7.3 (d) of ISO 15189:2022 states that “The EQA programme(s) selected by the laboratory shall, to the extent possible: 1) have the effect of checking pre-examination, examination, and post-examination processes; 2) provide samples that mimic patient samples for clinically relevant challenges; fulfil ISO/IEC 17043 requirements [24].

EQA schemes are now available that either look at pre-analytical or post-analytical elements alone or as part of an analytical EQA Scheme. These pre- and post-analytical elements can also be accredited to ISO/IEC 17043:2023 and participants must participate in the return of results if they are claiming ISO 15189:2022 accreditation in such areas [24]. Birmingham Quality asks for the interpretation of analytical results in a number of EQA Schemes, for example interpretation of the numerical B12, Ferritin and Folate in the UK NEQAS for Haematinic Assays and Vitamin D in the UK NEQAS for Vitamin D. Other examples of post analytical EQA schemes include the use of the analytical result reported by the laboratory within equations, for example eGFR or FIB4 and within algorithm pathways, AKI.

The types of EQA schemes discussed above describe elements that are present at every distribution. Educational EQA schemes also have the flexibility to include ad-hoc scenarios or surveys where the information reported is not used for any scoring elements but can be used to gain an understanding of current laboratory practices to provide evidence to laboratories and other stakeholders. This is the type of EQA that is presented in the current paper.

### Clinical context

Adrenal insufficiency occurs when the adrenal glands fail to produce adequate concentrations of primarily cortisol, and sometimes aldosterone. Cortisol is a glucocorticoid and is an essential hormone that affects almost every organ and tissue in the body. Cortisol is important as a stress response, to keep the body alert and ready to respond to danger. Cortisol also regulates the metabolism—how carbohydrates, fats and proteins are used; it modulates the immune system and plays a significant role in mood and cognitive function.

Cortisol is routinely measured by immunoassay and is available 24 h a day, 7 days a week by most, if not all laboratories. Cortisol is usually requested in cases of suspected adrenal insufficiency or cortisol excess (Cushings Disease). In both cases dynamic function tests, for example a short synacthen test (SST) (to stimulate the release of cortisol) or a dexamethasone suppression test (to suppress the release of cortisol) is performed with the cortisol results interpreted against local or standard guidelines.

Cortisol deficiency or insufficiency can have serious consequences and can be a medical emergency. Therefore, it is important to be able to reliably diagnose patients so that management plans can be put in place to allow them to live as normal a life as possible.

In August 2024, NICE released Guideline NG243 — “Adrenal insufficiency: identification and management” which provided guidance on interpretation of a single cortisol result in the context of suspected adrenal insufficiency [13].

The UK NEQAS for Steroid Hormones Scheme has provided an EQA service for cortisol for over 40 years. Serum specimens are provided for the analysis of only cortisol with the distribution of three specimens, notionally monthly to participants in the UK and worldwide. This Scheme design allows predominantly endogenous concentrations of cortisol to be distributed to laboratories. Hydrocortisone is added to some specimens to assess assay recovery and in some cases other steroids are added as part of interference studies, for example Prednisolone [25].

### Objectives

The aim of the current study was to look at the impact of both within- and between manufacturer/analyser variation when interpreting a single early morning serum cortisol against NICE guidelines — in this case NICE Guideline NG243 — “Adrenal insufficiency: identification and management” [13]. The EQA Scheme has been used to probe both the analytical and post-analytical aspects of this process based on a provided scenario with participants analysing three specimens designed to mimic potential adrenal insufficiency. All specimens contained endogenous concentrations of cortisol. The only manipulation was the pooling of donations to obtain the volume required, filtering of serum and two freeze-thaw cycles (preparation of specimens and distribution of specimens). Formal commutability studies have not been undertaken; however, we would assert that the material is commutable with patient material because filtering to 1 µm will not remove lipoproteins and serum cortisol has been shown to be very stable during at least four freeze thaw cycles [26]. There were no additions of exogenous cortisol, or dilutions made.

## Materials and methods

### Specimen preparation

Three pools (C663, C668 and C667) of serum were prepared using serum sourced as off-the-clot serum from NHS Blood and Transplant, Filton, UK. Serum was received frozen and used as “pooled material” with serum donations from donors with the same blood group and sex of donor being mixed and then filtered to 1 μm, using a glass fibre filter paper, Merck, Gillingham, UK. The maximum number of donations in each pool was eight. No exogenous material was added.

All three pools had previously been distributed through the EQA Scheme and the field method mass spectrometry mean was used to guide selection for Distribution 536 to align with required cut-offs for the proposed clinical scenario.

Each pool was pipetted into labelled 2 mL Sarstedt tubes (Nümbrecht, Germany) as 0.5 mL aliquots and stored frozen until dispatch where they were then transported at room temperature (the majority of specimens arrive within 24 h) 387 participants (82% UK and 18% overseas). Participants are advised to analyse immediately as if they were from a patient.

Data is also shown from other cortisol EQA specimens over the last year. All specimens are prepared according to the same protocol of using either single donations, or pooling together a number of donations, from the same sex donor, with the only exception being the addition of exogenous hydrocortisone (Merck Life Science Ltd., Gillingham, UK) as part of recovery experiments. These specimens are clearly identified to the participant as having been manipulated. No other additions are made unless intentional as part of an interference study — this data has not been included.

Data has been classified on whether the material is from a male or female donor and whether used endogenously (endog) or whether cortisol has been added (exog).

### Clinical scenario

A clinical scenario was provided with all three specimens:

The three Serum Cortisol specimens at Distribution 536 (A, B and C) comprise 3 different early morning (08:30 am) serum cortisol requests with a scenario ?Adrenal insufficiency.

You can assume the patients are 25-year-old females.

Please select the interpretation code from the dropdown:

Code 1 is Person may have adrenal insufficiency
Code 2 is Probability of adrenal insufficiency uncertain
Code 3 is Adrenal insufficiency very unlikely
Codes 4 and 5 were not applicable for Distribution 536

### Data analysis

For each specimen, participants reported back online (manually or automatically), to the Scheme at Birmingham Quality, the cortisol concentration measured. Participants are also able to check the method that they are using and can change if required to ensure that they are registered correctly.

Participants were also asked to interpret their cortisol result based on the clinical scenario and could choose one of 5 options from a drop-down menu.

Outliers in EQA schemes offered by Birmingham Quality are removed by Healy trimming with is applied to all data [27]. This essentially leads to the highest and lowest 5% of specimen level data being removed from statistical calculations of means and %CVS. A method mean, standard deviation and coefficient of variation is calculated for each manufacturer/analyser combination. The target value for every laboratory irrespective of the method that they are using is the field method mass spectrometry mean, which is calculated as the method mean of mass spectrometry users (which for Distribution 536 was based on 19 individual results). The field method mass spectrometry mean is routinely used as the target value for cortisol and has been validated by comparison of a number of pools with a candidate reference method for cortisol [28].

Each participant interpreted their measured cortisol result in terms of the clinical scenario provided. Data is presented for each specimen as (i) Rainbow Trout plot — a scatter plot for each method, coloured by interpretation, (ii) Pie chart — based on all reported cortisol interpretations and (iii) Bar chart — split by method and presented both as number of reported results and percentage of each method. The target value for each interpretation is based on the interpretation of the field method mass spectrometry cortisol mean in relation to the cut-offs provided within NICE Guideline NG243 — “Adrenal insufficiency: identification and management” [13].

Data has also been reviewed and is presented for each of the four devolved nations within the UK.

## Results

### Cortisol analysis

**FIGURE 1:**
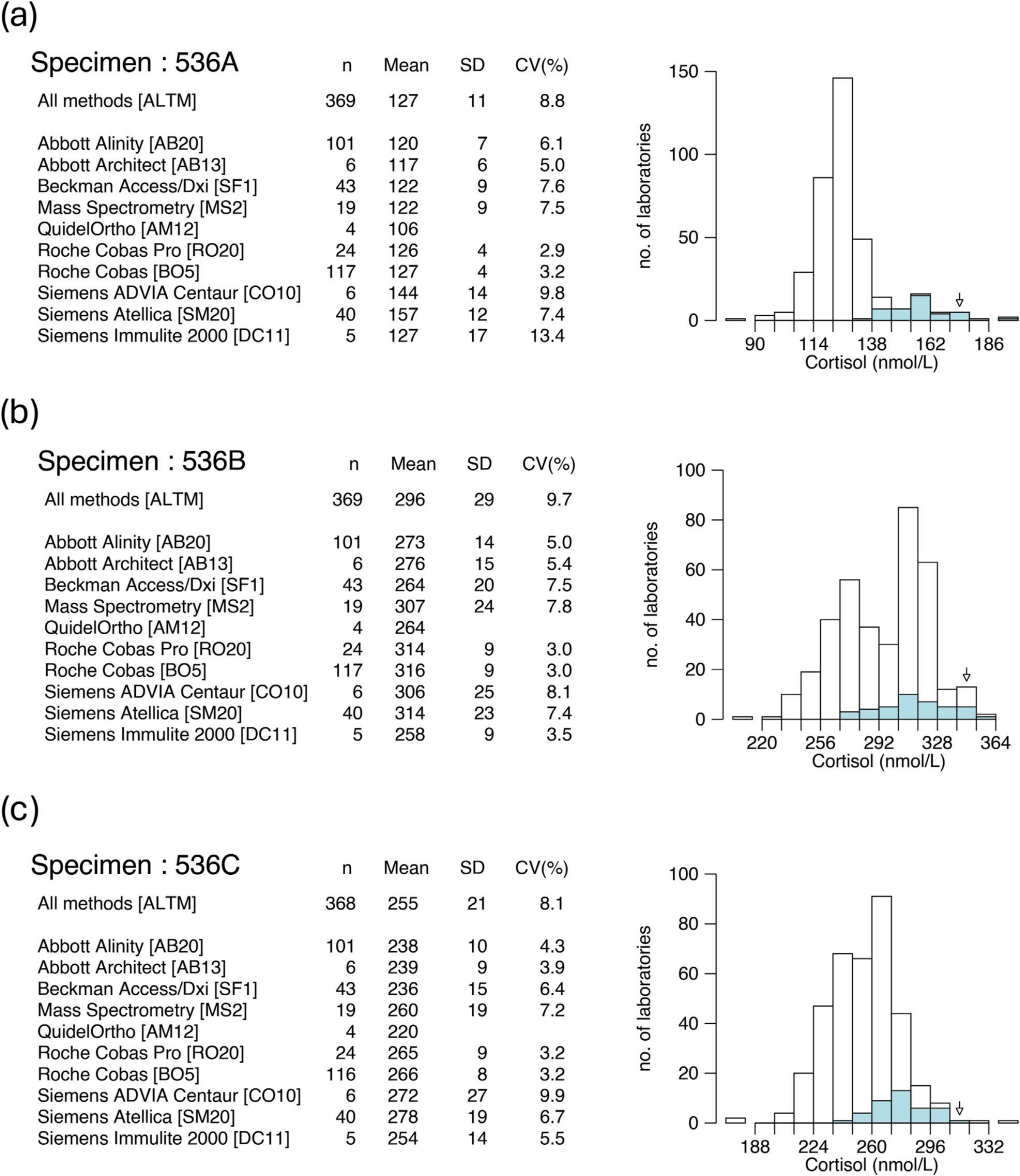
Histograms and associated manufacturer/analyser data for **(a)** Specimen 536A, **(b)** Specimen 536B and **(c)** Specimen 536C. The blue section represents Siemens Atellica cortisol data (n = 40).

**FIGURE 2:**
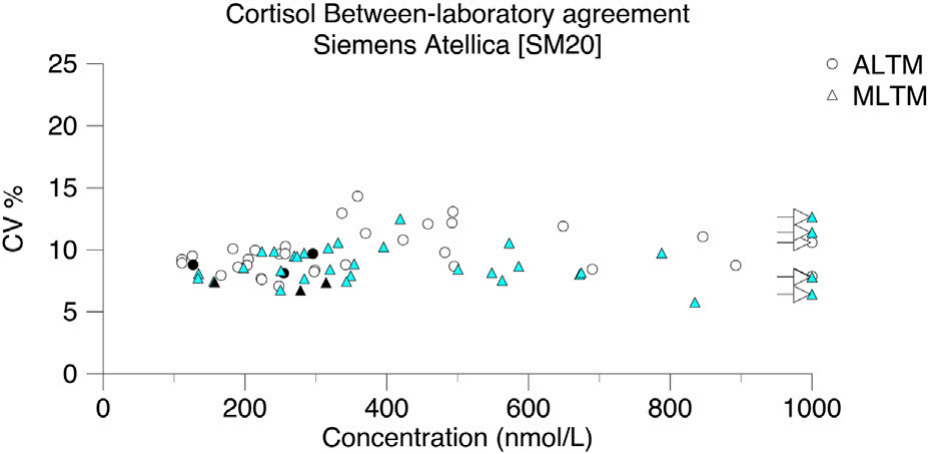
Between-laboratory-agreement plot for Cortisol showing the %CV against concentration for 36 individual specimens over the last 12 distributions. Data is shown for the All Laboratory Trimmed Mean (ALTM) as open circles — this is the average of all data after Healy Trimming has been applied to the raw cortisol results — and data is also shown for the Method Laboratory Trimmed Mean (MLTM) as green triangles, which in this figure is for the Siemens Atellica — this is the average of all data from the Siemens Atellica method group, after Healy Trimming has been applied. The black circles and triangles correspond to data for the current distribution — specimens 536A, 536B and 536C.

In December 2025 results, cortisol results were returned by ∼370 participants in the UK NEQAS for Steroid Hormones Scheme (Distribution 536). The breakdown by manufacturer/analyser is shown in Figure 1 with the Roche Cobas and the Abbott Alinity being the two most used methods. Figure 1 shows histograms and associated tabulated data for the three specimens at Distribution 536. The target value is the field method mass spectrometry mean which for Specimen 536A is 122 nmol/L (SD 9 nmol/L, %CV 7.5%), Specimen 536B is 307 nmol/L (SD 24 nmol/L, %CV 7.8%) and Specimen 536C is 260 nmol/L (SD 19 nmol/L, %CV 7.2%). These three specimens have behaved exactly as expected at this point in time and there are no concerns regarding specimen homogeneity or stability, as seen by the %CVs (∼10%) which are similar to distributions of previously distributed material at similar concentrations (Figure 2). Stability and homogeneity are checked on every specimen, at every distribution. Degradation of a specimen during the preparation stage, or whilst in transit, would show a wider spread of reported results which consequently would impact the standard deviation and corresponding %CV.

**FIGURE 3:**
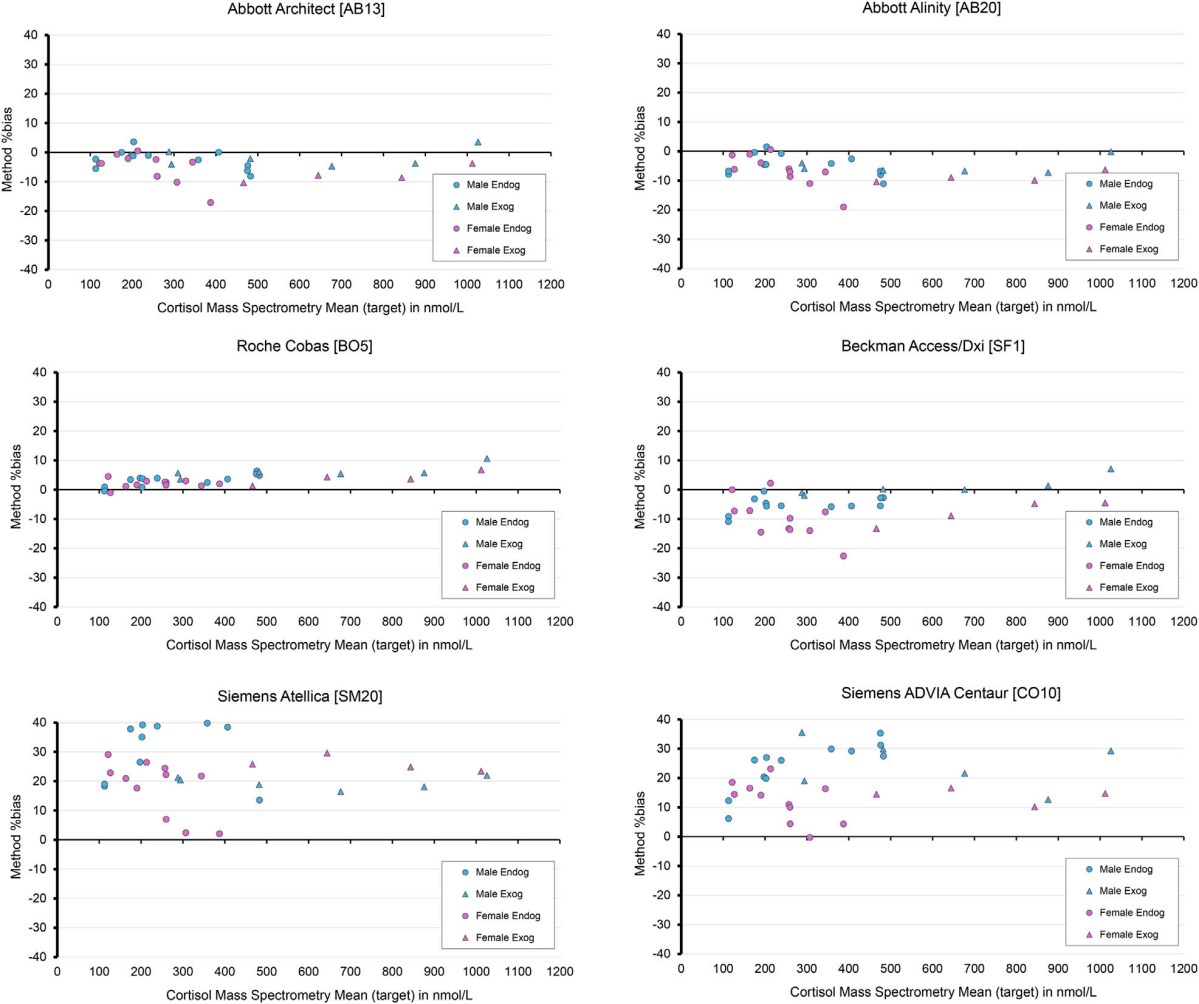
Individual specimen %biases compared to the target value, the field method mass spectrometry mean, for each manufacturer/analyser as detailed in each chart. Data is shown by sex of donor contributing to the pool (blue is male, pink is female) and whether the pool contained endogenous concentrations of cortisol (circle) or whether exogenous cortisol was added (triangle). The data shown is for the time period January 2025 to December 2025.

Commutability with a patient sample has not been proven, but there is nothing to suggest that using off-the clot serum with no manipulation other than the pooling of a number of donations, filtering to 1 µm and two freeze thaw cycles would cause commutability issues. Single donations would be preferred; however, this does not allow for repeat testing which is a valuable tool in the assessment of whether the performance of a method has changed. There are different relative method biases across the three specimens, at different cortisol concentrations; however, these are as expected and are consistent with what has previously been seen — see Figure 3.

Figure 3 shows the individual specimen % biases compared to the target value, the field method mass spectrometry mean for all data for the major manufacturers between January and December 2025. The Roche Cobas cortisol method is minimally biased with respect to the target value and there appears to be no difference in bias between male and female specimens. In comparison, both the Siemens ADVIA Centaur and Siemens Atellica cortisol assays show a much wider spread of biases and there is a clear difference between male and female matrices.

### Cortisol interpretation

**FIGURE 4:**
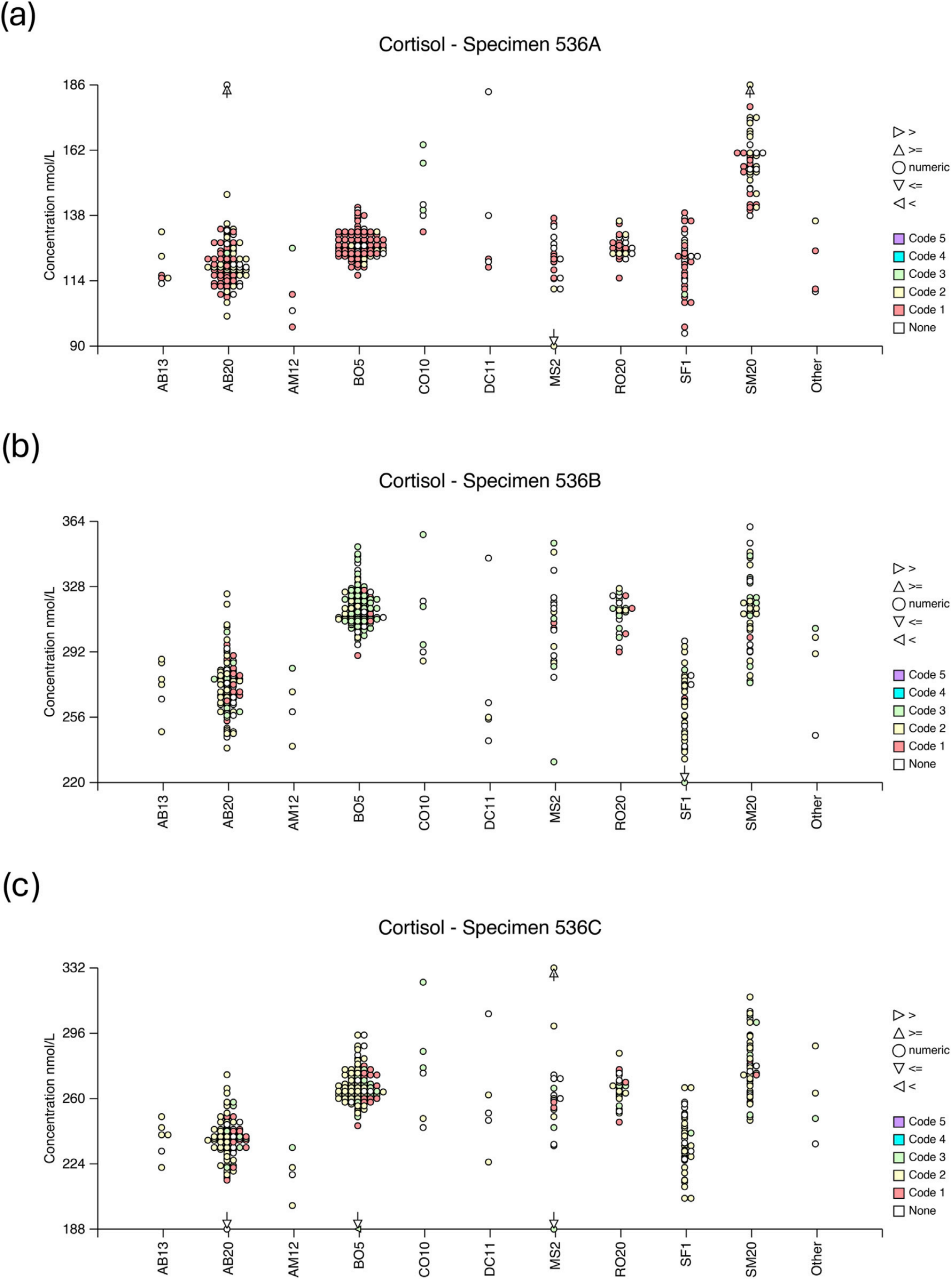
Scatter plots (Rainbow Trout plots) for **(a)** Specimen 536A, **(b)** Specimen 536B and **(c)** Specimen 536C. Code 1 = Person may have adrenal insufficiency, Code 2 = Probability of adrenal insufficiency uncertain and Code 3 = Adrenal insufficiency very unlikely. The bar charts show the interpretation coding based on the binned concentration as per NG243. The coding on the x-axis relates to the method code — *AB13 = Abbott Architect, AB20 = Abbott Alinity, AM12 = QuidelOrtho, BO5 = Roche Cobas, CO10 = Siemens ADVIA Centaur, DC11 = Siemens Immulite 2000, MS2 = Mass Spectrometry, RO20 = Roche Cobas Pro and SM20 = Siemens Atellica*.

**FIGURE 5:**
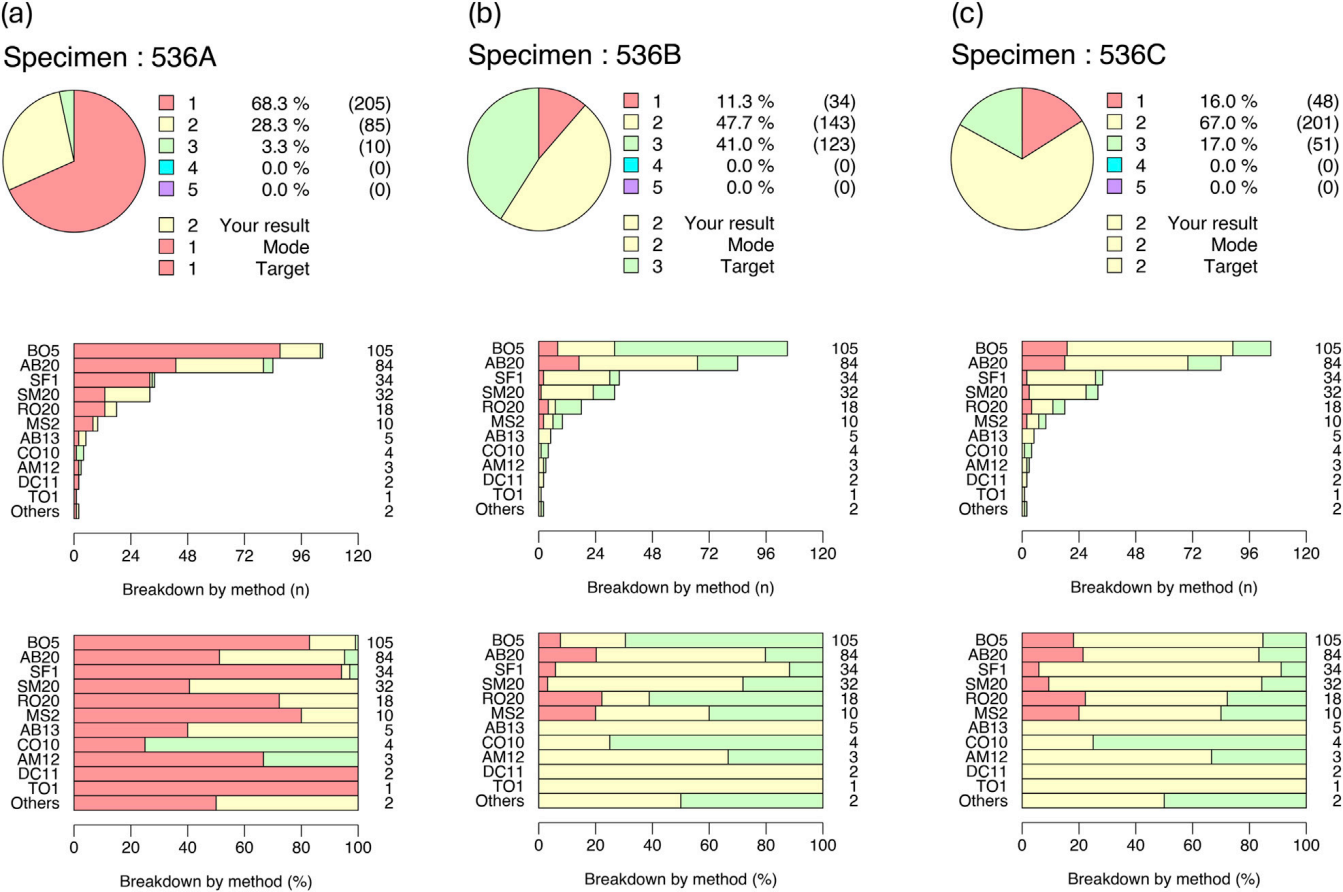
Pie and bar charts for interpretation of all specimens in relation to the provided clinical scenario for **(a)** Specimen 536A, **(b)** Specimen 536B and **(c)** Specimen 536C. Code 1 = Person may have adrenal insufficiency, Code 2 = Probability of adrenal insufficiency uncertain and Code 3 = Adrenal insufficiency very unlikely. The bar charts show the interpretation coding based on the binned concentration as per NG243. The coding on the bar charts relates to the method code — *AB13 = Abbott Architect, AB20 = Abbott Alinity, AM12 = QuidelOrtho, BO5 = Roche Cobas, CO10 = Siemens ADVIA Centaur, DC11 = Siemens Immulite 2000, MS2 = Mass Spectrometry, RO20 = Roche Cobas Pro, SM20 = Siemens Atellica and TO1 = Tosoh.*

Participants were asked to interpret their measured cortisol result with the scenario that the sample had been taken from a 25-year-old female at 08:30 am. The scenario did not make reference to guidelines. This was intentional because the aim was to assess how laboratories interpret based on their usual procedures, not to report a response based on available guidelines. Interpretations were returned by ∼300 participants (82% of the total number of participants reporting numerical cortisol results). Figures 4, 5 show the same cortisol interpretation data but presented in a different way. Figure 4 is the Birmingham Quality trademark Rainbow Trout plot (scatter plot) which shows each individual cortisol result (y-axis) by method (x-axis) colour coded based on the interpretation provided by the Laboratory itself (see legend). This data presentation shows quite clearly the spread of results, by method, and that some method/manufacturers, for example Roche (BO5) for Specimen 536A has a smaller spread of user results than for example Beckman (SF1), for the same specimen. Also, the Siemens Atellica (SM20) not only has a similar spread of results to Beckman, but nearly all results are greater in value than Roche. Data is presented as a scatterplot where up to 8 identical values are plotted on a single row then on another row above for a further 8. This visualisation nicely shows that not only is there variation in interpretation between manufacturers, but also within a manufacturer. This can be seen by the different colour filled circles within the same concentration bin.

Figure 5 shows a summary of all data, in a pie chart, for all three specimens broken down by interpretation. Data is only included for categories 1, 2 and 3 because those are the only codes relevant for this exercise. The bar charts below the pie charts break down interpretation by method/manufacturer. The bottom bar chart is the same data as the top bar chart normalised as a percentage. For Specimen 536A, which had a cortisol of 122 nmol/L, 68% of responses were Code 1 with a significant proportion reported by all manufacturers. The mode was taken as the Target Value. However, Specimen 536B, which had a cortisol of 307 nmol/L had a more even split of interpretations (between code 2 and code 3). For Specimen 536B the cortisol concentration is known to be 307 nmol/L based on the field method mass spectrometry mean and has been interpreted based on Nice Guideline NG243. This cortisol concentration corresponds to code 3, which is what has been set as the Target Value, not the mode, which was code 2. A number of participants, from six methods, reported Specimen 536B as code 1. The majority of interpretations were split between codes 2 and 3. For Specimen 536C, which had a cortisol of 260 nmol/L, 67% of responses were code 2. In this case the mode was the assigned Target Value. Interestingly, participants from the same six methods as Specimen 536B, reported a Code 1 for the interpretation of Specimen 536C.

**FIGURE 6:**
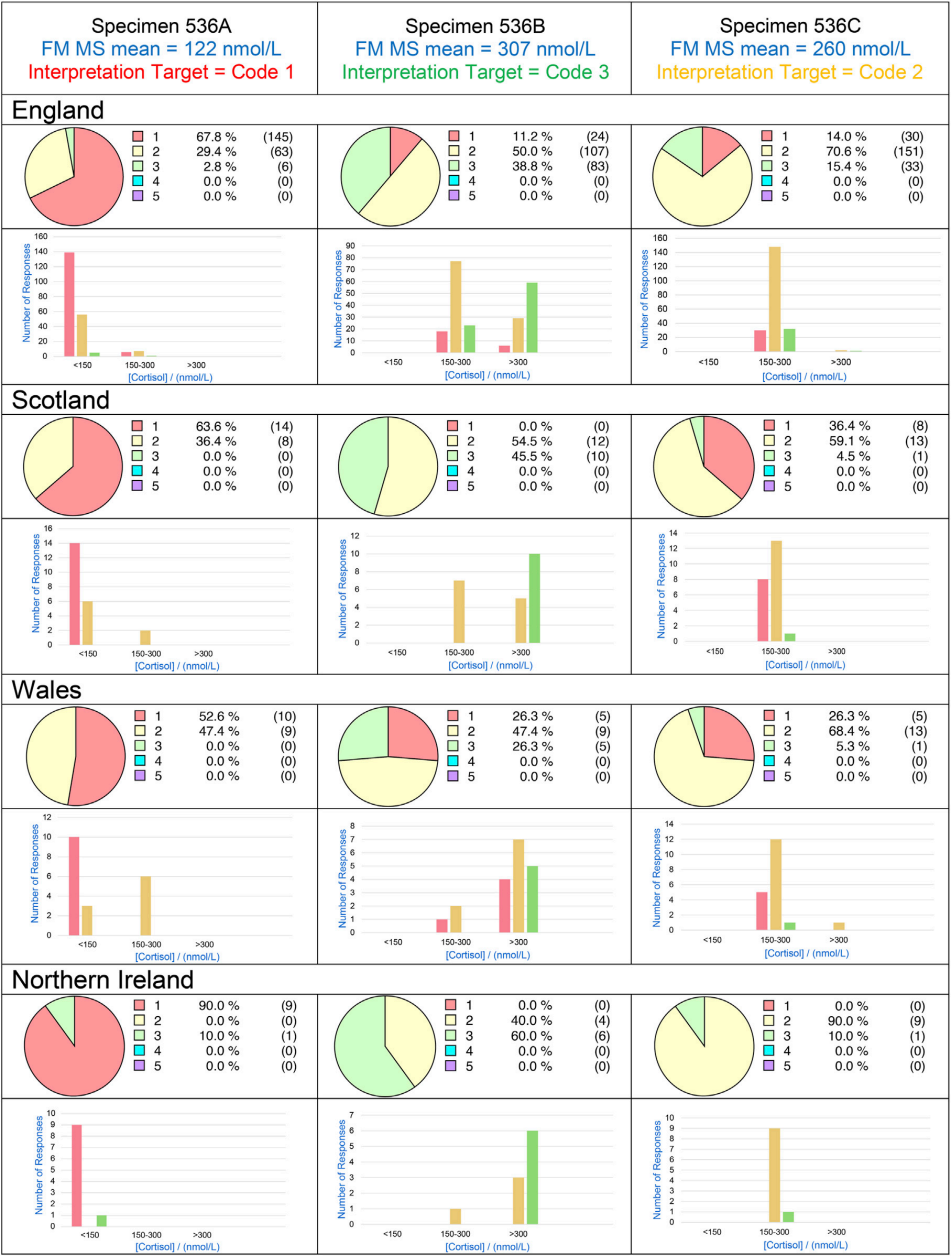
Breakdown of Cortisol interpretation for each specimen, by country and also by cortisol concentration. Code 1 (Red) = Person may have adrenal insufficiency, Code 2 (Orange) = Probability of adrenal insufficiency uncertain and Code 3 (Green) = Adrenal insufficiency very unlikely. The bar charts show the interpretation coding based on the binned concentration as per NG243.

Data has been broken down by each laboratory in each devolved nation within the UK (Figure 6). Pie charts are shown for each specimen, for each data set, as previously discussed. Data has been further broken down by cortisol concentration bin <150 nmol/L, 150–300 nmol/L and >300 nmol/L which corresponds to the cortisol concentration bins used in Nice Guideline NG243. There are no obvious trends within a region or for a specific specimen.

Participants are able to add comments to all their EQA returns and a number were made specifically for cortisol at this distribution. A number are detailed in Table 1 and relate to two themes (i) requirement for further information and (ii) comments applied.

## Discussion

The medical laboratory’s role is more than the provision of a numbers only service to clinicians. The majority of UK clinical laboratories will provide some form of clinical interpretation of the result whether this is an auto comment (which may be specific to the reported numerical result), a manual comment by a “duty biochemist,” addition of additional tests (reflex or reflective) or may be clinical advice given over the phone if contacted by a clinician [29, 30]. This is the “added value” that laboratories can provide to their service and is highly valued.

Test results are usually compared to reference ranges (reference intervals) or decision limits but for a number of disease states national/international guidelines exist, or local guidelines may be in place. NICE Guidance is evidence-based recommendations for the health and social care sector, developed by independent committees, including professionals and lay members, and consulted on by stakeholders [5]. NICE guidelines are aimed at healthcare in England; however, they are available to be used by anyone.

In the area of adrenal insufficiency. NICE Guideline NG243 — “Adrenal insufficiency: identification and management” was produced in August 2024. The guidance is to offer an 8 am–9 am serum cortisol test to people aged 1 year and over with suspected adrenal insufficiency. Table 1 of NG243 details the three concentration bins for cortisol with advice for people aged 16 years and over and children and young people between 1 year and over and under 16 years. The three cortisol concentration bins are <150 nmol/L, 150–300 nmol/L and >300 nmol/L.

The three concentrations of cortisol chosen for this study corresponded with the three binned concentrations and for the purposes of the EQA interpretation three codes were used corresponding to the definitions of each concentration bin:

Code 1 is Person may have adrenal insufficiency

Code 2 is Probability of adrenal insufficiency uncertain

Code 3 is Adrenal insufficiency very unlikely

This article is not a detailed review of the analytical performance of cortisol; however, it is useful to know how methods perform at different cortisol concentrations and in male and female matrices. As expected a wide spread of cortisol results was returned for each specimen, both in terms of known method/manufacturer specific concentration dependent biases and also sex related differences in cortisol measurement for some assays.

Variation in interpretation based on the clinical scenario of the specimens being taken from a 25-year-old female at 08:30 am was also observed for each specimen. From Figure 4, there is not a clear concentration cut-off for these different interpretations within a single method which suggests that different guidelines/protocols are being used to interpret a single cortisol result in a clinical scenario of ?Adrenal insufficiency. NICE Guidance is aimed at English hospitals, but 56 (29%) of English laboratory responses have interpreted cortisol concentrations <150 nmol/L as code 2 (Probability of adrenal insufficiency uncertain) and 5 (3%) reported code 3 (Adrenal insufficiency very unlikely). Therefore, looking at England alone, it is clear that NICE Guidance is not fully being followed. This may be because some laboratories wanted more information—clinical state of the patient, were they on the oral contraceptive pill or pregnant etc. Or it may be because of differences in guidance protocols, or no protocols being in use. Whatever the reason is it is clear that there is unwarranted variation which ultimately will impact patient safety either directly due to the consequences of a delay in diagnosis or emotional/psychological damage through mis-diagnosis. The consequences to the NHS are rarely quantified; however, it is clear that there will be a financial impact which could then further impact other patients.

Data review for this exercise has been limited to comparison to NICE NG243; however, there are other guidelines and resources available for the investigation of suspected adrenal insufficiency, for example the Endocrine Society Guidelines which recommends the use of an SST as the optimal diagnostic test for adrenal insufficiency [31]. The standard dose of 250 ug corticotrophin stimulation is recommended with peak cortisol levels below 500 nmol/L at 30 or 60 min indicating adrenal insufficiency. The Endocrine Society Guidelines do include a comment that this peak cortisol concentration of 500 nmol/L is assay dependent. There are a number of publications which have tried to correlate the between assay differences and provide assay specific cortisol cut-offs for the interpretation of an SST. The most notable of which is a 2013 paper by N. El-Farhan on Method-specific serum cortisol responses to the adrenocorticotrophin test: comparison of gas chromatography-mass spectrometry and five automated immunoassays [32]. Cortisol concentration assay dependent biases and sex-dependent differences continue to make this a complicated area.

Differences between cortisol assays have been known for over three decades. In 1991 JG Middle reported that there were clear differences between methods for cortisol [33]. In 2010 the first UK audit of the short synacthen test showed that the variability in bias of the cortisol methods had not been translated to the cut-off values used by the majority of laboratories [16]. Metrological traceability of laboratory assays is at the forefront of a number of discussions. This current paper was not intended to be a full review of cortisol assays; however, it is clear to see from the limited data that has been presented that there are still differences between manufacturers and some assays allow more accurate and precise measurement than others. For example, the Roche Cobas cortisol assay gives comparable results to the field method mass spectrometry method mean across the concentration range 100–1100 nmol/L for both sexes, whereas both the Siemens ADVIA Centaur and the Siemens Atellica show a wider spread of biases (indicating a less precise assay) and sex dependent biases. Sex-related issues continue to be an issue for some manufacturers. At the time of writing this paper Siemens are in the process of implementing a Control System Improvement which is aimed at improving accuracy and precision through improvements to lot-to-lot consistency. We are hopeful that improvements in EQA data will be seen over the coming months. There is a wider spread of biases for both Abbott cortisol assays and the Beckman cortisol assay compared to Roche Cobas which would indicate precision issues potentially from lot-to-lot variability.

The Roche Cortisol II assay was designed for improved specificity and our EQA results show that there is very good evidence of this with good correlation to the field method mass spectrometry target value for both male and female serum. It was well recognised at the time of introduction that cortisol results were 20%–30% lower than its predecessor and 50-year old SST cut-off values (based on poor specificity assays) should be adjusted (500 or 550 nmol/L values often used) [34]. However, unfortunately sometimes these “magic numbers” are engrained into the mindset of clinicians and though assays change, it is harder to change the habit of the clinician. This is the same for most if not all tests where common reference ranges have been adopted.

We are aware that Siemens are in the process of implementing a Control System Improvement for their cortisol assay and some/all participants will be in the process of moving over to this, hence the improvement in performance.

An Editorial by JW Honour in 2017 reported *“Huge sums of money are spent on quality assurance of assay performances yet external quality assurance (EQA) programmes have failed for decades to use a powerful evidence-base of the poor quality of assays to put pressure on assay suppliers to improve accuracy and specificity of tests …”* [34] As expected there was rebuttal from EQA providers at the time [35, 36]; however, can the EQA provider be expected alone to take on this role? It is a sad, but true fact that some laboratories view EQA providers merely as a “supplier” of a service that allows them to gain and then maintain their ISO 15189:2022 accreditation. The EQA providers that are used within the UK are mainly departments within NHS hospitals and therefore do not have the legal infrastructure to tackle head on the multi-million-pound manufacturers. EQA providers like Birmingham Quality have been showing through their schemes the evidence that there are differences between manufacturers/assays and in some cases wide variation within a single method. UK laboratories have always had the evidence, but they have continued to sign into multi-million pound managed service contracts without themselves challenging manufacturers.

The governance structure of EQA is currently “paused” and has been since 1st January 2026 [37]; however, prior to 1st January 2026 the “hosts,” the Royal College of Pathologists were also not in a position to legally challenge manufacturers [38]. In “The Missing Piece,” Marrington et al. compared the current governance structure within the UK to a jigsaw which was impossible to complete — not only because a piece was missing, but because the pieces did not fit together properly [39].

Nearly 10 years after that initial editorial, it is hoped that we may now be in a position to better use the evidence from high quality EQA [40], to drive change which has a positive impact on patient safety [23].

We are not advocating that all laboratories must follow NICE Guidance. The purpose of this study was to show that there are variations in clinical chemistry assays resulting in variation in the value of the number reported. We have shown that there is unwarranted variation for cortisol assays. This is not a new finding and embarrassingly shows something that all laboratories will have been aware of for the last three decades at least.

Laboratories and the authors of guidelines need to be aware of this variation (across all methods/manufacturers) which could potentially be used to generate test results which could then later be applied to a guideline with a single cut-off (as in NG243). This unwarranted variation is not limited to cortisol. A number of clinical biochemistry (and potentially other laboratory assays) are impacted, and it is incumbent on the laboratory to communicate the limitations of assays to clinicians even if the system in use in their laboratory is not impacted. It is usually clinicians that are driving improvement initiatives and without awareness of how assays perform then algorithms or online calculators are appearing with good intentions, which could ultimately cause more harm to the patient.

This work represents an advance in biomedical science because EQA is being shown to be more than assessment of an analytical result. Incorporating post-analytical assessment variation in interpretative practices can be identified.

### Limitations

This study required participants to analyse and interpret cortisol result as part of their routine EQA. Neither element is mandatory. Conclusions have been formed on the best data set that we have available; however, we know that because this is EQA, and not a patient sample, it may have been different personnel who were interpreting results than those who would a patient sample.

Cortisol data has been linked to manufacturer/method information. We try to ensure that it is correct; however, we are completely reliant on the Participant informing us of any changes to the system that they are using.

## Summary table

### What is known about this subject

- EQA has always shown variation in cortisol assays — concentration dependent and sex related biases
- Laboratory data is being used in “big data” sets to develop new derived tests and algorithms
- Lack of documentation of the impact of assay variation is hindering the drive for improvements in analytical assays

### What this paper adds

- Post-analytical EQA has been used to show the impact of the variation in cortisol assays across different manufacturers
- Awareness of the limitations of assays which should be taken into consideration when interpreting test results
- Adds to the evidence base of cortisol assay performance and clinical requirements which can now be used as part of post market surveillance

## Data Availability

The original contributions presented in the study are included in the article/supplementary material, further inquiries can be directed to the corresponding author.
